# Linking ER Stress to Autophagy: Potential Implications for Cancer Therapy

**DOI:** 10.1155/2010/930509

**Published:** 2010-01-17

**Authors:** Tom Verfaillie, Maria Salazar, Guillermo Velasco, Patrizia Agostinis

**Affiliations:** ^1^Department of Molecular Cell Biology, Cell Death Research and Therapy Laboratory, Faculty of Medicine, Catholic University of Leuven, 3000 Leuven, Belgium; ^2^Department of Biochemistry and Molecular Biology I, School of Biology, Complutense University, 28040 Madrid, Spain; ^3^Centro de Investigación Biomédica en Red sobre Enfermedades Neurodegenerativas (CIBERNED), 28040 Madrid, Spain

## Abstract

Different physiological and pathological conditions can perturb protein folding in the endoplasmic reticulum, leading to a condition known as ER stress. ER stress activates a complex intracellular signal transduction pathway, called unfolded protein response (UPR). The UPR is tailored essentially to reestablish ER homeostasis also through adaptive mechanisms involving the stimulation of autophagy. However, when persistent, ER stress can switch the cytoprotective functions of UPR and autophagy into cell death promoting mechanisms. Recently, a variety of anticancer therapies have been linked to the induction of ER stress in cancer cells, suggesting that strategies devised to stimulate its prodeath function or block its prosurvival function, could be envisaged to improve their tumoricidial action. A better understanding of the molecular mechanisms that determine the final outcome of UPR and autophagy activation by chemotherapeutic agents, will offer new opportunities to improve existing cancer therapies as well as unravel novel targets for cancer treatment.

## 1. Introduction

The endoplasmic reticulum (ER) is an organelle with crucial biosynthetic and signaling functions in eukaryotic cells. The ER is not only the major intracellular calcium (Ca^2+^) storage organelle critically involved in Ca^2+^ homeostasis and Ca^2+^ mediated signaling pathways, but it also provides the environment for the synthesis, folding, and modification of proteins destined to be secreted or embedded in the plasma membrane (reviewed in [1, 2]). Moreover, the ER is the major site for the biosynthesis of steroids, cholesterol, and lipids. Proper folding, maturation, and stabilization of the nascent protein in the ER require the highly oxidizing and Ca^2+^-rich ER environment, which is essential for the diverse posttranslational and cotranslational modifications, including glycosylation and disulfide bridge formation, to which proteins are subjected after entering the ER. These processes are assisted and monitored by several resident chaperones and Ca^2+^ binding proteins, including the glucose-regulated proteins [such as GRP78 or BiP (immunoglobulin heavy-chain binding protein)], calreticulin and calnexin, and several folding enzymes, such as the thioredoxin-like protein disulfide isomerase (PDI). PDI oxidizes cysteine residues in nascent proteins (i.e., oxidative folding) resulting in formation of intra- and intermolecular disulphide bonds, while reduced PDI is in turn oxidized by the thiol oxidoreductase ERO1. ERO1 transfers reducing equivalents to molecular oxygen, generating stoichiometric amounts of H_2_O_2_ per newly formed disulphide, which is coupled with a depletion of the reduced gluthatione pool [3]. Proteins that fail to adopt a correctly folded or native conformation, or a proper oligomeric assembly in case of multisubunit proteins, are retrotranslocated to the cytosol through a process known as ER-associated protein degradation (ERAD), and further degraded by the 26S proteasome.

Various physiological and pathological conditions, including hypoxia, ER-Ca^2+^ depletion, oxidative injury, high-fat diet, hypoglycemia, and viral infections may cause an imbalance between ER protein folding load and capacity, leading to the accumulation of unfolded proteins in the ER lumen, a condition referred to as “ER stress”. ER stress sets in motion an evolutionary conserved and integrated signal transduction pathway known as the Unfolded Protein Response (UPR). The UPR primarily aims at ameliorating the protein load on the ER by coordinating the temporal shut down in protein translation along with a complex program of gene transcription to increase ER folding capacity. If this transcriptional program fails to reestablish proper ER homeostasis, persistence in ER stress induces cell death. 

Severe ER stress can cause cell death, usually by activating intrinsic apoptosis [4]. Moreover, in order to clear the ER from the accumulation of terminally misfolded protein aggregates that cannot be degraded by the proteasome, the UPR may upregulate the autophagy machinery [5, 6]. Macroautophagy (hereafter referred to as autophagy) is a major lysosomal pathway for the in bulk degradation of cytoplasmic materials, including proteins and damaged organelles, characterized by the sequestration of entire portions of the cytoplasm by a double-membrane bounded vacuole called the autophagosome [7, 8]. In spite of its role as a self-digestion mechanism, autophagy is mainly activated to protect against cell death [8]. However, just like in the case of the UPR, stimulation of autophagy can under certain circumstances be required to activate the cell death machinery [9]. Although both the UPR and autophagy can function independently from each other, recent reports show that they may be interlinked and share the functional duality of exerting both a cytoprotective (under basal or metabolic stress conditions) and cytocidial activity (after acute cellular damage). 

Tumor cells are bathed in a hostile microenvironment and confronted with chronic metabolic stress conditions that favor the activation of adaptive mechanisms, such as the UPR and autophagy [10, 11].Moreover, certain promising anticancer regimens have been shown to activate concomitantly ER stress and autophagy in cancer cells (see Section 4). The molecular link between the UPR and the autophagic response to ER stress, and how these stress pathways influence therapeutic outcome, remain largely undefined, making this topic a very important area for future research in cancer therapy. 

Here, we review the molecular mechanisms underlying the emerging connections between the UPR and autophagy pathways, and discuss their potential implications in the context of anticancer therapy. 

## 2. Signal Transduction in ER Stress 

### 2.1. UPR Signaling Pathways

The unfolded protein response in mammalian cells is governed by three transmembrane ER stress sensors, namely PERK (PKR-like ER kinase), IRE1 (inositol requiring enzyme 1), and ATF6 (activating transcription factor 6), which are kept in an inactive state by binding to the ER chaperone BiP, preventing their oligomerization-induced activation. When ER homeostasis is perturbed, accumulating misfolded proteins become progressively bound to BiP, titrating away BiP from interaction with these transmembrane signaling proteins. Upon deinhibition and homodimerization these ER sensors activate a complex ER-to-nucleus signaling pathway that transmits information across the ER membrane to an extensive gene-expression program mediated by the activation of downstream transcription factors. The genetic program activated by the UPR results in upregulation of the folding machinery along with an expansion of the ER lumen and enhanced degradation of terminally misfolded proteins through ERAD. Additional mechanisms include a general translational shutdown as well as the degradation of a select group of secretory mRNAs and proteins that are delayed at the translocon, a process also known as pre-emptive quality control [12]. The mechanisms by which UPR induction coupled with the failure in reestablishing the ER folding capacity leads to cell death and the requirement of UPR signaling in autophagy stimulation, are still unsettled questions. Furthermore, recent studies have revealed that the ER serves as a subcellular platform for the formation of signaling complexes comprising molecular elements of the UPR, Bcl-2 family members (both pro- and antiapoptotic) (reviewed in [13]), and perhaps regulators of autophagy. 

**Figure 1:**
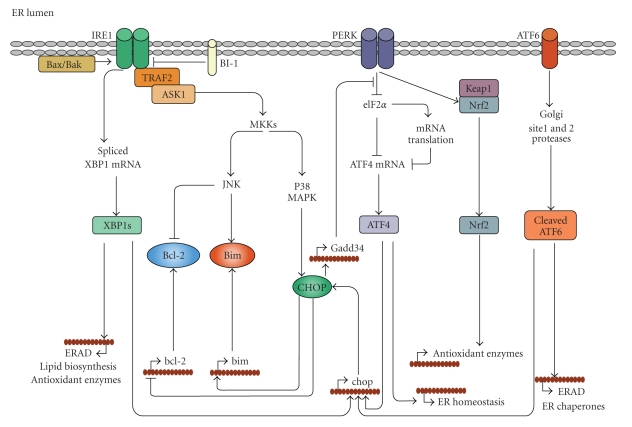
The unfolded protein response in adaptive, apoptotic and redox responses. Upon accumulation of misfolded proteins in the ER, the release of BiP allows IRE1 and PERK to oligomerize. Oligomerized IRE1 disposes of an intrinsic endoribonuclease activity that mediates the unconventional splicing of XBP1mRNA which is subsequently translated into XBP1s, a potent transcription factor regulating expression of genes involved in ERAD and ER quality control. IRE1 signaling is positively regulated by binding of the multidomain proapoptotics Bax and Bak, while its activity is suppressed by the transmembrane protein BI-1. The interaction of Bax/Bak with IRE1 is required for the recruitment of TRAF2 and ASK1 leading to the activation of the MAPKs JNK and p38 MAPK, through specific MKKs. Oligomerized PERK phosphorylates the translation initiating factor eIF2*α*, resulting in suppression of general protein translation while favoring the translation ATF4, which induces the expression of genes involved in restoring ER homeostasis. Phosphorylation of Nrf2 by PERK disrupts its association with Keap1 resulting in its nuclear accumulation and upregulation of genes associated with various antioxidant responses. In contrast to PERK and IRE1, release of BiP from ATF6 induces its translocation to the Golgi where its processing generates an active transcription factor. Cleaved ATF6 controls mainly genes involved in ERAD and ER homeostasis. Upon severe ER stress, ATF4, XBP1s, and ATF6 can upregulate the expression of the proapoptotic transcription factor CHOP, which mediates apoptosis by the upregulation of proapoptotic BH3-only protein Bim and by suppressing Bcl-2 expression. CHOP activity is enhanced through phosphorylation by p38MAPK. Phosphorylation by JNK in turn activates Bim while inhibiting Bcl-2 functions.

In the following sections we will discuss current knowledge on the main signaling pathways emanating by each branch of the UPR along with their downstream targets (Figure 1). 

#### 2.1.1. IRE1

Two human isoforms (paralogous) of yeast Ire1 have been identified. IRE*α* is expressed in all cell types and tissues, whereas expression of IRE1*β* is primarily restricted to the epithelial cells of the gastrointestinal tract [14, 15]. IRE1 is a type I transmembrane protein with an N-terminal luminal sensor domain and a C-terminal cytosolic effector region that contains both kinase and endoribonuclease (RNase) domains [16]. In cells undergoing ER stress, oligomerization of IRE1 results in trans-autophosphorylation and activation of the RNase domains which excise a 26 nt sequence from *XBP1u *(unspliced *XBP1*), producing mature *XBP1s* mRNA (spliced *XBP1*) [17]. *XBP1s* encodes an active leucine zipper (bZIP) transcription factor XBP1s that regulates the transcription of several genes involved in ER quality control mechanisms, ER/Golgi biogenesis, as well as ERAD components [18–22] and as recently revealed, also genes involved in redox homeostasis and oxidative stress responses [23]. 

Consistent with this, XBP1 deficient cells were found to be more susceptible to exogenous agents causing oxidative stress, such as H_2_O_2_ and parthenolide, concomitant with a reduced expression of several antioxidant enzymes including catalase and thioredoxin (TRX1) [24]. Overexpression of XBP1 restored catalase expression and reduced ROS generation after H_2_O_2_, thus implicating a protective role for XBP1 in oxidative stress. Intriguingly, this antioxidant effect was mediated by XBP1u (i.e., the protein encoded by the unspliced XBP1 mRNA), whereas XBP1s (i.e., the product of IRE1 activation) failed to induce changes in catalase expression in response to ROS or following ER stress, thus underscoring that IRE1 activity is dispensable [24]. Although the molecular mechanism underlying the differential function of the unspliced (XBP1u) and spliced (XBP1s) products of XBP1 is still elusive- and may involve the binding and regulation of selected targets dependent on their relative abundance in different cell types [23] this study highlights a new role for XBP1 in ROS signaling, independent of IRE1 RNase activity. 

Although IRE1 displays intrinsic kinase activity, there are no other substrates known thus far than IRE1 itself. However, prolonged activation of IRE1 is capable to transmit a MAP kinase activation cascade. It has been shown that IRE1 can serve as a molecular platform for the recruitment of the adaptor protein TNF-receptor associated receptor 2 (TRAF2), an E3 ubiquitin ligase, which leads to the activation of apoptosis signal regulating kinase 1 (ASK1), a MAP3K of the JNK/p38 MAPK pathway [25, 26]. Depending on the cellular context, activation of JNK can either allow the cells to adapt to ER stress by initiating autophagy [5] or, as discussed further, promote apoptosis in response to persistent or irrecoverable ER stress.

#### 2.1.2. PERK

Like IRE1, PERK is a type I transmembrane protein with a luminal sensing domain and a cytosolic kinase domain which becomes activated following dimerization. The resulting transautophosphorylation induces a conformational change that enhances the affinity of PERK for eIF2*α* (eukaryotic initiation factor 2 alpha) [27]. Phosphorylation of eIF2*α* on Ser51 by PERK results in the rapid shut down of general translation, relieving the protein burden on the stressed ER, while the concomitant loss of cyclin D1 arrests the ER stressed cells in G1. In addition, a recent study has shown that eIF2*α* phosphorylation also regulates translation via inhibition of rRNA synthesis, coordinately regulating translation and ribosome biogenesis during cellular stress [28]. Paradoxically, this translational shutdown leads to the selective translation of the transcription factor ATF4, a member of the bZIP family of transcription factors [29]. The PERK-eIF2*α*-ATF4 axis regulates the expression of genes involved in amino acid biosynthesis and transport functions, antioxidant stress responses, and apoptosis.

In addition to eIF2*α*, PERK also phosphorylates the Nuclear factor-E2-related factor 2 (Nrf2) [30]. Nrf2 is a bZIP Cap ‘n Collar transcription factor that integrates a variety of cellular responses to oxidative stress. Nrf2 is maintained inactive in a cytoplasmic complex with the microtubule associated protein KEAP1 (Kelch-like Ech-associated protein 1). Nrf2 phosphorylation promotes its dissociation from KEAP1, leading to the nuclear accumulation of Nrf2, binding the antioxidant response element (ARE) in the promotor of genes encoding detoxifying enzymes such as heme oxygenase 1 (HO-1) [31]. In line with these results, it was shown that Nrf2^−/−^ cells are more prone to ER stress induced apoptosis [30]. Likewise, PERK^−/−^ cells, along with an impaired attenuation of protein synthesis, were found to mount a high amount of endogenous peroxides preceding apoptotic induction in response to agents causing perturbation of ER functions [32]. Interfering with ERO1 blocked the increased ROS production, thus providing a link between protein oxidation in the ER and ROS production during ER stress. This PERK function was linked to the ability of ATF4 to regulate the expression of genes involved in glutathione biosynthesis and antioxidant response [32]. These studies suggest that the PERK branch of UPR bifurcates in two parallel but integrated signaling pathways, PERK-eIF2*α*-ATF4 and PERK-Nrf2, with a key role in adaptation to oxidative stress, a metabolic consequence of biosynthesis and posttranslational oxidative processing in the ER.

#### 2.1.3. ATF6

Both isoforms of ATF6, ATF6*α* and ATF6*β*, are present in all cell lines as type II transmembrane ER proteins. Release of BiP does not cause ATF6 oligomerization, but instead reveals a Golgi localization sequence [33]. Once translocated to the Golgi, ATF6 is cleaved at a juxtamembrane site by the site 1 and site 2 proteases (S1P and S2P) [34], which are also involved in the cleavage of the ER membrane transcription factor SREBP (sterol response element-binding protein) [35, 36]. Processed ATF6 moves to the nucleus where it forms active homodimers or dimerizes with other bZIP transcription factors like NF-Y (CAAT binding factor) as well as XBP1s [37], to regulate transcription from ATF/cAMP response elements (CREs) and ERSEs [38;39]. One of the ATF6 transcriptional targets is the IRE1 substrate *XBP1* [22]. Interestingly, Yoshida *et al. *found that XBP1u interacts directly with the active form of ATF6 (but not ATF4), targeting it for proteasomal degradation which may provide a negative feedback loop to decrease XBP1 expression [38]. Other transcriptional targets include proteins increasing ER chaperone activity and degrading of ER client proteins [37, 39]. Although ATF6 is neither essential for basal expression of ER chaperones nor for embryonic or postnatal development, it plays an important role in recovery from acute ER stress and adapting cells to chronic ER stress [39]. Additionally, a recent study shows that ATF6*α* also contributes, in an XPB1s-independent manner, to lipid biogenesis and ER expansion, an ER stress response which was thought to be predominantly mediated by the IREI pathway [40].

### 2.2. Regulation of the UPR by Bcl-2 Family Members

Bcl-2 family proteins, which consist of proapoptotic multidomain proteins (e.g., Bax, Bak), antiapoptotic multidomain proteins (e.g., Bcl-2), and BH3-only proteins (e.g., Bid, Bim, Bad), are key regulators of mitochondrial apoptosis [41, 42]. They function as gatekeepers (antiapoptotic; Bcl-2) or gatecrashers (proapoptotic; Bax/Bak) of the outer mitochondrial membrane [43]. While the molecular mechanism underlying their mitochondrial action is still a matter of debate, it is becoming clear that Bcl-2 family proteins can exert a tight control on apoptosis at different subcellular sites. A constellation of ER localized Bcl-2 family members, including Bax, Bak, Bik/Nbk, and Bcl-2, has been shown to be engaged in the control of ER-Ca^2+^ homeostasis [44–46] (for an extensive review see [13, 47]). Moreover, recent reports have identified Bcl-2 members as vital regulators of UPR sensor mechanisms and cellular fate following ER stress. 

For instance, ATF6 negatively regulates Bad proapoptotic activity by upregulating regulator of calcineurin 1 (RCAN1) [48], an endogenous inhibitor of calcineurin (protein phosphatase B). Bad dephosphorylation allows its dimerization with antiapoptotic Bcl-2 protein family members like Bcl-X_l_, thus inhibiting their activity [49]. This mechanism underscores a prosurvival role for the genetic program activated by ATF6, through the suppression of Bad proapoptotic activity [49].

The proapoptotic multidomain proteins Bax and Bak can form a protein complex with the cytosolic domain of IRE1, and this interaction has been shown to be essential for IRE1 signaling [50]. Genetic ablation of *bax/bak* in mice caused abnormal response to tunicamycin-induced ER stress in the liver along with extensive tissue damage, decreased expression of XBP1, and reduced JNK activation [50]. Furthermore, the requirement of Bax/Bak proteins for proper IRE1 signaling was confirmed in MEFs doubly deficient (DKO) in these proapoptotic proteins [50]. In a recent report Klee et al. [51] showed that reconstituting Bak expression at the ER membranes in DKO cells is sufficient to reestablish IRE1-TRAF2 activation and mitochondrial apoptosis (as discussed further in Section 2.3) instigated by reticular forms of the BH3-only proteins Bim and Puma. Interestingly, the IRE1 pathway activated by reticular BH3-only effectors was atypical as it did not lead to XBP1 splicing, likely because other arms of the UPR required for the upregulation of *XBP1* mRNA levels, such as ATF6, were not sufficiently activated [51]. However, an alternative and intriguing possibility could involve a differential regulation of the IRE1 RNase activity (required for *XBP1 *mRNA splicing) and IRE1-TRAF2 complex formation (required to activate proapoptotic JNK signaling) by a different subset of proapoptotic proteins at the ER membrane. Clearly, further studies are required to shed more light into the mechanisms regulating IRE1 signal transduction. 

Recently, the ER associated Bax inhibitor-1 (BI-1), an evolutionary conserved antiapoptotic protein, has been identified as a new player in the regulation of IRE1 by Bcl-2 family members and their modulators. BI-1 can block Bax-mediated apoptosis following ER stress and other intrinsic stress signals by directly interacting with antiapoptotic Bcl-2 family members and enhancing their antiapoptotic function [52]. BI-1 at the ER was found to be capable to interact through its C-terminus domain with IRE1 and to inhibit IRE1 signaling, in vitro as well as in mice and flies, conferring increased resistance under conditions of mild ER stress [53]. ER stressed BI-1 deficient cells displayed IRE1 hyperactivation along with an increased *XBP1* mRNA splicing and expression of XBP1s-dependent genes, thus unraveling a paradoxical role of BI-1 as inhibitor of the cytoprotective IRE1 branch of the UPR in mildly ER stressed cells. Interestingly, in another study using human fibrosarcoma cells, overexpression of BI-1 inhibited ROS production downstream ER stress through the upregulation of HO-1, an effector of the PERK-pathway (as described previously) [54]. Although in the study of Lisbona et al. [53] BI-1 deficiency in MEFs did not affect expression of HO-1, this raises the intriguing possibility that BI-1 may affect IRE1 and possibly PERK pathway in a cell type specific manner. Whereas further mechanistic studies are required to solve these discrepancies, these findings reveal how a subtle cross-talk between molecular sensors of UPR and cell death regulators might affect the amplitude and function of the UPR. Moreover, abundant evidence claims for a critical role of proapoptotic Bcl-2 family proteins in the induction of apoptosis following ER stress.

### 2.3. From ER Stress to Cell Death

When the initial cellular responses fail to restore ER homeostasis, sustained ER stress causes the UPR to switch from an adaptive to a cell death pathway. However, the molecular elements of this switch are still elusive. With the exception of few components of the UPR for which a dominant prosurvival (i.e., BiP, [55]) or proapoptotic (i.e., CHOP, [56, 57]) role has been assigned by genetic studies, each apical UPR sensor holds a dualistic role in propagating adaptive as well as a toxic signals. 

For example, genetic deletion of *PERK* or interference with eIF2*α* phosphorylation impairs cell survival [58, 59] and tumor growth under hypoxia [60], while artificially increasing PERK activity increases cell survival [61]. However, Lin et al. [62] have shown that sustained PERK signaling is lethal, whereas the equivalent duration of IRE1 signaling is not, suggesting that transition from protective to proapoptotic UPR function involves a switch in IRE1 signaling along with enduring PERK activity [62].

The main effector of PERK-mediated apoptosis is the proapoptotic transcription factor CHOP (C/EBP homologous protein; GADD153) which can be induced by ATF4, ATF6, as well as XBP1s. However, the PERK-eIF2*α* branch appeared to be essential for CHOP upregulation as both PERK^−/−^, ATF4^−/−^ and eIF2*α* Ser51Ala knock-in cells failed to induce CHOP during ER stress [32, 58, 59]. CHOP activity is also regulated translationally by the limited CHOP mRNA lifetime [63] and posttranslationally by p38MAPK phosphorylation, which enhances its proapoptotic activity [57, 64]. The latter mechanism may provide a point of convergence between the PERK and IRE1 signaling pathways since p38MAPK is a downstream target of the IRE1-TRAF2-ASK1 signaling complex [25, 26]. Genetic studies have shown that *CHOP *loss-of-function results in cytoprotection, whereas *CHOP* gain-of-function enhances sensitivity to a variety of stresses perturbing ER function [56, 65]. 

CHOP mediated cell death entails the induction of a variety of genes that may potentiate apoptosis, including *GADD34*, * ERO1* *α* *, Bim*, and *TRB3* (Tribbles homologue 3). GADD34 is a regulatory subunit of protein phosphatase 1 (PP1) that targets PP1 to dephoshorylate eIF2*α*, which promotes the resumption of protein synthesis [66]. If the protein folding capacity of the ER has not been reestablished, a premature deinhibition of translation will increase client protein load in the ER and may favor improper disulphide bond formation of unfolded proteins, thus amplifying the damage. In addition, elevated expression of ERO1*α* by CHOP is thought to instigate hyperoxidizing conditions in the ER [67, 68]. Thus the PERK-axis, which is involved in maintaining the redox state during ER stress, as discussed before, has also the ability to turn into a prooxidant signal when the transcriptional program of CHOP is efficiently set in motion. 

As suggested by a recent study wherein the stability of prosurvival and prodeath mRNAs and proteins was studied under conditions of mild or severe ER stress [63], ATF4-dependent prosurvival gene expression is likely to be more sustained when PERK is activated transiently and to a limited extent. In contrast, as a consequence of the intrinsic instability of the proapoptotic mRNAs and proteins, the apoptotic program mediated by the ATF4 target CHOP would be activated only when protective mechanisms fail and require a more sustained PERK activation. 

CHOP can also regulate the expression of a number of Bcl-2 family proteins. By a yet unidentified mechanism, it suppresses the expression of the antiapoptotic Bcl-2 [65] while directly promoting the transcription of the proapoptotic BH3-only protein Bim [69]. 

Although it is clear that CHOP fulfills an important role in ER stress induced apoptosis, the fact that PERK^−/−^ and eIF2*α* Ser51Ala knock-in cells are unable to induce CHOP yet are very susceptible to ER stress [58, 59] unravels the dual role of the PERK axis in triggering both adaptive and proapoptotic processes. The increased sensitivity of PERK deficient cells could be explained, at least in part, by the impaired activation of the prosurvival PI3K (phosphatidylinositol 3 kinase)-Akt signaling pathway which has been shown to promote the expression of inhibitor of apoptosis proteins (IAPs), thus conferring cellular resistance to ER stress [70, 71]. 

An interesting molecular switch between the prosurvival and prodeath functions of the PERK pathway could involve the human orthologue of the *Drosophila* tribble protein (TRB3), a downstream transcriptional target of CHOP [72]. Ohoka and et al. [72] showed that TRB3 knock down sensitized the cells to cell death during tunicamycin treatment. Remarkably, TRB3 could downregulate its own induction by repressing CHOP/ATF4 functions [72, 73]. A mechanism was proposed wherein TRB3 exerts a negative feedback on CHOP during mild ER stress, allowing the cell to adapt to ER stress [72, 73]. In contrast, during severe or persistent ER stress, induction of TRB3 would be more robust, leading to apoptosis through a mechanism involving TRB3-mediated inhibition (dephosphorylation) of Akt [74, 75]. This feedback mechanism could facilitate ER stress mediated apoptosis in severely ER stressed cells that have successfully mounted proapoptotic threshold levels of CHOP.

Similar to PERK, IRE1 signaling has also been implicated in promoting or impairing cell survival. For instance, when unfolded proteins accumulate, artificially extending IRE1's RNase function led to enhanced survival [62, 76] and the knock down of XBP1 impaired cell survival, [77, 78] pointing to a general protective role for the IRE1-XBP1 signaling during ER stress. However, in another report, IRE1 overexpression in HEK293T cells led to its activation in the absence of ER stress and subsequent cell death [14]. As discussed before, IRE1 has apparently gained signaling properties independent of XBP1 splicing, which are strongly dependent on interaction with Bcl-2 proapoptotics and Bcl2 modulators at the ER membrane. Thus, IRE1 can promote cell death by recruiting a TRAF2-ASK1 complex leading to the activation of JNK and p38 MAPK cascades [25, 26]. JNK, in turn, can exert its proapoptotic effect by activating certain BH3-only proteins, such as Bim [79, 80], or by suppressing the antiapoptotic activity of Bcl-2 [81]. 

The apoptotic pathway evoked after UPR is still unclear, but mounting observations indicate that the mitochondrial pathway is heavily involved, since cells lacking Bax and Bak, or Apaf-1 are resistant to apoptosis induction by different ER stressors [44, 82, 83]. Moreover, as mentioned before, several Bcl-2 family members localize at the ER and regulate both calcium levels as well as signal transduction through the UPR. In addition to Bim, other BH3-only proteins, such as Noxa and Puma, are transcriptionally activated, through p53-dependent [84] and independent mechanisms [85] depending on the type of ER stressor, thus bridging ER stress to Bax/Bak mediated mitochondrial membrane permeabilization. Recently Klee and coworkers using Bax^−/−^/Bak^−/−^ cells showed that Bak targeted at the ER membrane is sufficient to engage mitochondrial apoptosis when activated by BH3-only molecules Puma and Bim at the ER, thus bypassing the need to be localized to the mitochondria [51]. Reticular Bak engaged an atypical IRE*α*-TRAF2 activation pathway, wherein the mobilization of Ca^2+^ facilitated persistent JNK activation [51]. Intriguingly, ER Ca^2+^ release *per se* was not able to incite mitochondrial apoptosis unless Bak was expressed at the reticulum [51], whereas it favored nonapoptotic cell death, as shown also in our previous study [82]. Whether this pathway has any role in normal cells expressing both mitochondrial and ER Bax/Bak still needs to be proven, however it can already be argued that JNK functions as a master regulator of both apoptosis and perhaps autophagy pathways after ER stress. 

Thus all together the emerging consensus is that the amplitude and the temporal activation of specific arms of the UPR, along with the repertoire of signaling platforms formed at the ER membrane (UPR interactome), are crucial elements determining cellular fate following ER stress.

## 3. ER Stress and Autophagy

### 3.1. Autophagy

Proteasomal degradation and autophagy are the two main mechanisms that are in charge of protein clearance in the cell. Unlike proteasomal degradation (that digests soluble ubiquitin-conjugated proteins in a specific way), autophagy can degrade both soluble and aggregated proteins [8, 86]. Thus, during the autophagic process, entire cytoplasmic portions—including organelles and other cytoplasmic components—are engulfed within a double membrane vesicle designated autophagosome. The maturation of these vesicles involves their fusion with lysosomes, which leads in turn to the degradation of the autophagosome components by the lysosomal degradative enzymes [8, 86]. As discussed below, a variety of stress signals such as nutrient starvation or treatment with different anticancer agents (including those that induce ER stress) stimulate the autophagy process—which is nowadays considered as an essential cellular process participating in a number of physiological functions within the cell.

The molecular mechanisms responsible for the regulation of autophagy have not been completely elucidated yet, although genetic and biochemical analyses performed during the last few years have identified several autophagy genes (Atg) that participate in the regulation of this cellular process. Researchers working in the autophagy field have formally divided the autophagic process in several steps. Initiation of autophagy relies on the formation of an isolation membrane (IM) at the so-called preautophagosomal site. Elongation of this isolation membrane leads to the formation of the autophagosome. The autophagy process ends with the fusion of the autophagosome and the lysosome, the digestion of the autophagosome content, and the release of the digested components back to the cytosol [8, 86]. In these sections, we will briefly summarize the mechanisms by which the different stages of autophagy are regulated.

The normal rate of autophagy in the cell is low and therefore this cellular process only becomes activated in response to certain situations. Thus, exposure of the cell to an autophagic stimulus triggers a series of modifications in the autophagic machinery that allow the formation and elongation of the IM. The precise origin of the IM in mammalian cells is still unknown, although it has been proposed that it could be either derived from *de novo* synthesized lipids or generated by vesicle budding from ER, Golgi apparatus, or endosomes [87]. The transmembrane proteins Atg9 and VMP-1 [88, 89] are required for autophagosome formation and it has been suggested that they could play a role in the transport of lipids to the IM as well as in the recruitment of additional proteins involved in the initiation of autophagy. Thus, the movement of Atg9 from the trans-Golgi location to the preautophagosomal site seems to be a crucial event in the initiation of autophagy [87, 90].

The relocation of the transmembrane protein Atg9 to the autophagosome is thought to require activation of the complex formed by the proteins Atg1, Atg13, and Atg17/FIP200.[88].The activity of the Atg1 complex is modulated by the mammalian target of rapamycin complex 1 (mTORC1). mTORC1 is a protein complex formed by mTOR, RAPTOR (regulatory associated protein of mTOR), mLST8, and PRAS40 (proline-rich AKT substrate 40 kDa) [91] that plays a central role in the control of protein synthesis, cell growth, and cell proliferation through the regulation of several downstream targets [91]. In addition, mTORC1 has been proposed to regulate autophagy by repressing the activity of the Atg1-Atg13-Atg17/FIP200 complex [92–95]. Thus, inhibition of mTORC1 facilitates the initiation of autophagy. Regulation of mTORC1 relies on the small G protein Rheb (ras homologue enriched in brain) which (through a still not completely elucidated mechanism) activates mTORC1. The tuberous sclerosis proteins (TSC1 and TSC2) have GTPase activating protein (GAP) activity on Rheb and therefore promote its inhibition. Hence, inactivation of TSC1/2 stimulates Rheb and mTORC1 and inhibits autophagy [91]. 

As a result of its central position in the control of cellular homeostasis, mTORC1 integrates signals from different inputs. One of the most important upstream regulators of mTORC1 is the prosurvival kinase Akt, which phosphorylates and inactivates TSC2 as well as PRAS40 [91]. Thus, Akt activation stimulates mTORC1 and inhibits autophagy. Another important regulator of TSC2 is the AMP-activated protein kinase (AMPK) which phosphorylates TSC2 in a different residue than Akt leading to activation of TSC1/2, inactivation of Rheb, and inhibition of mTORC1 [96]. As discussed in the following sections, modulation of mTORC1 activity is one of the mechanisms by which ER stress and autophagy become connected.

Another important step in the initiation of autophagy is the generation of a specific pool of phosphatidylinositol-3-P (PIP3) at the autophagosome. In mammals, this event is catalyzed by the class III phosphatidylinositol 3 kinase (PI3K) complex [which consists of Vps34 (vacuolar protein sorting 34) and its regulatory protein p150 (homolog to the yeast Vps15 protein)] [97]. Accumulation of PIP3 seems to be crucial for the recruitment of autophagy proteins such as Atg18/WIPI-1 to the IM which is important for Atg9 trafficking and therefore for the initiation of the autophagic process [90]. In addition, other proteins such as mAtg2 and DFCP1 (double FYVE domain-containing protein 1) may also be regulated by PIP3 and play a role in the regulation of the formation and elongation of the autophagosome [90]. Underlining the importance of PIP3 in the early stage of autophagy, a specific phosphoinositide 3-phosphatase (Jumpy) has been very recently identified as a new modulator of this cellular process [98].

Importantly, other Vps34-interacting proteins are required for autophagy including, Vps30/Atg6/Beclin1, Atg14 and autophagy/beclin-1 regulator 1 (Ambra-1), and UVRAG [86]. Among the different partners of Vps34, particular attention has been focused on Beclin-1. Beclin-1 has a BH3-only domain that permits the interaction of this protein with the antiapoptotic proteins Bcl-2 and Bcl-X_L_. This interaction abrogates Beclin-1 ability to induce autophagy [99–102]. Different stimuli, including ER stress, modulate the interaction between Beclin-1 and Bcl-2 family members (see also the following sections) which is considered an important mechanism of autophagy regulation.

Atg14 and UVRAG are also interactors of Vps34 although their presence in the class III PI3K complex seems to be mutually exclusive [87, 103, 104]. Recent findings support that Atg14 plays an important role in the early stages of autophagy activation in response to starvation [87]. In any case, further research is still necessary to understand the complex lipid-protein and protein-protein interactions that regulate the formation of the IM.

 The elongation of the initial autophagic membrane requires the participation of two ubiquitin-like protein conjugation systems which modify the autophagy proteins Atg5 and Atg8/LC3. Thus, upon autophagy stimulation, Atg5 is conjugated to Atg12. In this process Atg12 is activated by the E1 activating enzyme Atg7 and transferred to the E2-like protein Atg10. Finally Atg12 is attached to an internal lysine of Atg5 in a process that does not seem to require an E3 ligase protein. The Atg5-Atg12 conjugation complex interacts with Atg16L to form the Atg16L complex [87]. The other conjugation system involves the modification of Atg8/LC3. Initially Atg8/LC3 is cleaved by the protease Atg4 (which generates a glycine Ct residue in Atg8/LC3). Then, the E1 enzyme Atg7 activates Atg8, which is transferred to the E2-like protein Atg3. The last step in Atg8/LC3 modification involves the conjugation of this protein to phosphatidylethanolamine (PE), a process that is facilitated by the E3-like activity of the Atg12-Atg5 conjugate [105, 106]. Upon autophagy induction, most of Atg8/LC3 becomes lipidated and associates with the autophagosome, which is widely used to monitor activation of autophagy by immunofluorescence [8, 86, 107]. The Atg16L and Atg8/LC3 complexes play a crucial role on the modification of the autophagosomal membrane and therefore in the elongation and closure of the autophagosome.

The last step in the autophagic process is the fusion of the autophagosome with lysosomes. The canonical machinery of vacuole membrane fusion seems to participate in the regulation of this process [87, 90]. Thus, the lysosomal protein LAMP2 and the small GTPase Rab7 have been implicated in autophagosome-lysosome fusion in mammalian cells. Nevertheless, many additional proteins including those belonging to the Rab and soluble N-ethylmaleimide sensitive factor attachment protein receptors family (SNARE) are believed to play an important role in the autophagosome-lysosome fusion process [8, 86]. The lysosomal degradation of the autophagosomal content relies on several lysosomal hydrolases including cathepsins B, D, and L.

The final outcome of the activation of the autophagy program is highly dependent on the cellular context and the strength and duration of the stress-inducing signals. Thus, besides its role in cellular homeostasis, autophagy can be a form of programmed cell death or play a cytoprotective role, for example in situations of nutrient starvation [108, 109]. Accordingly, autophagy plays a dual role in cancer. On one hand, this cellular process may help to overcome the stress evoked by the lack of nutrients and oxygen at the initial steps of tumorigenesis. On the other hand, autophagy has been proposed to play a tumor suppressor function by providing the minimal supply of ATP required for DNA repair, preventing oxidative stress and reducing intratumoral necrosis and local inflammation [110–113]. Moreover, different anticancer treatments activate autophagy in tumor cells, which has been proposed to either enhance cancer cell death or act as a mechanism of resistance to chemotherapy [110–115].

### 3.2. Connecting ER Stress Responses and Autophagy

**Figure 2:**
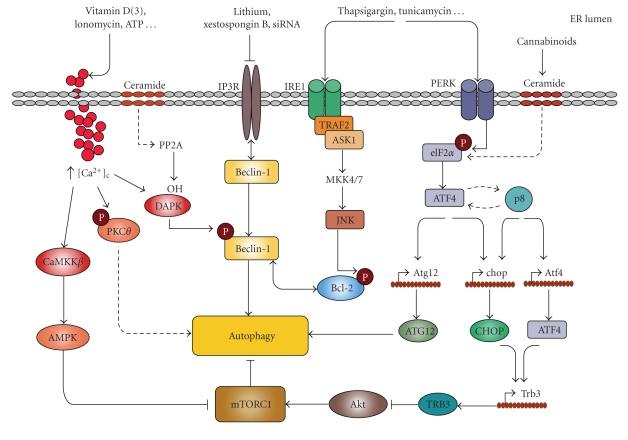
Mechanisms connecting ER stress and autophagy. Different ER stresses lead to autophagy activation. Ca^2+^ release from the ER can stimulate different kinases that regulate autophagy. CaCMKK*β* phosphorylates and activates AMPK which leads to mTORC1 inhibition; DAPK phosphorylates Beclin-1 promoting its dissociation from Bcl-2; PKC*θ* activation may also promote autophagy independently of mTORC1. Inositol 1,4,5-trisphosphate receptor (IP3R) interacts with Beclin-1. Pharmacological inhibition of IP3R may lead to autophagy in a Ca^2+^-independent manner by stimulating its dissociation from Beclin-1. The IRE1 arm of ER stress leads to JNK activation and increased phosphorylation of Bcl-2 which promotes its dissociation from Beclin-1. Increased phosphorylation of eIF2*α* in response to different ER stress stimuli can lead to autophagy through ATF4-dependent increased expression of Atg12. Alternatively, ATF4 and the stress-regulated protein p8 promote the up-regulation of the pseudokinase TRB3 which leads to inhibition of the Akt/mTORC1 axis to stimulate autophagy.

Different situations that induce ER stress also lead to induction of autophagy. As discussed above, the ER stress response is activated to protect the cells from different alterations affecting this organelle. However, when the intensity or duration of the ER damage cannot be restored by this response, ER stress can also lead to cell death [116]. Likewise, autophagy can help cells to cope with ER stress (for instance contributing to the elimination of unfolded or aggregated proteins) or participate in the mechanism of ER stress-induced cell death [115, 117–119]. In this section we will try to delineate some of the proposed mechanisms by which ER stress and autophagy become connected under certain cellular situations (Figure 2). 

#### 3.2.1. UPR and Autophagy

As described above, the accumulation of unfolded proteins triggers the UPR thus promoting the inhibition of general protein synthesis as well as the increased translation of several transcription factors that enhance the expression of ER stress genes [117] (see the previous sections for further details). Evidence for a link between UPR and autophagy was obtained from ectopic expression of polyglutamine (polyQ) proteins [6]. In these experiments, a dominant-negative form of PERK or genetic substitution of Serine 51 of eIF2*α* by Ala (which prevents the phosphorylation of this protein) prevented polyQ protein-induced autophagy [6], strongly suggesting that PERK-dependent eIF2*α* phosphorylation plays an important role in the activation of autophagy in response to the accumulation of unfolded proteins. On the other hand, eIF2*α* phosphorylation seems to be also important for autophagy as induced by other ER stress-related or unrelated stimuli [75, 120, 121]. It is important to bear in mind that PERK is not the only protein kinase regulating eIF2*α* phosphorylation (see reference [116] for a review) as double-stranded RNA-activated protein kinase (PKR; activated in viral responses), general control nonderepressible 2 (GCN2; activated upon aminoacid starvation), and heme-regulated inhibitor (HRI; activated in heme depletion) also phosphorylate eIF2*α*. Accordingly, PKR-dependent eIF2*α* phosphorylation modulates autophagy in response to viral infection [120]. Likewise, the small heat shock 22 KDa protein 8 (HspB8) and its cochaperone Bcl-2-associated athanogene 3 (Bag3) have been proposed to mediate mutated hungtingtin-induced eIF2*α* phosphorylation and autophagy via GCN2 activation [121]. 

Regarding the signalling pathways by which eIF2*α* phosphorylation can modulate autophagy, Kouroku and et al. showed that PERK-eIF2*α*-dependent Atg12 upregulation is required for induction of autophagy in response to polyQ protein accumulation [6]—which suggests that controlling the expression of autophagy-related genes by eIF2*α* downstream targets could be one of the mechanisms connecting both events. On the other hand, we have recently found that treatment of cancer cells with Δ^9^-tetrahydrocannabinol (THC), the active component of marihuana, activates autophagy via ER stress and eIF2*α* phosphorylation [75] (an effect that is not mediated by PERK, PKR, or GCN2, Salazar, M. and Velasco, G. unpublished observations). Our data indicate that induction of autophagy in response to THC treatment relies on the eIF2*α* phosphorylation-dependent upregulation of the transcription factors p8, ATF-4, and CHOP as well as of the pseudokinase TRB3 (four genes that had been previously identified as essential mediators of THC action in cancer cells [122, 123]). We also showed that an important step in the induction of autophagy is the inhibition of the Akt/mTORC1 axis by the pseudokinase TRB3 [75] (see below for additional details) (Figure 2). In any case, further research is still necessary to clarify the precise mechanisms by which eIF2*α* phosphorylation regulates autophagy in response to different ER stress signals.

Activation of the IRE1 arm of the ER stress response has also been shown to regulate autophagy. Thus, treatment with tunicamycin or thapsigargin [5] or treatment with proteasome inhibitors [119] induced autophagy on an IRE1-dependent manner. The proautophagic actions of IRE1 seem to rely on the ability of this protein to interact with the cytosolic adaptor TRAF-2 and activate JNK) [5]. Of interest, JNK has been proposed to regulate autophagy through Bcl-2 phosphorylation, which prevents this protein of interacting (and inhibiting) the essential autophagy regulator Beclin-1 [99, 124, 125]. In addition, JNK has been shown to control Beclin-1 expression to regulate ceramide-induced autophagy [126]. As discussed above, Beclin-1 is associated to the Vps34 and plays a very important role in the regulation of autophagy ([102]see below) (Figure 2). It is therefore conceivable that activation of the IRE1/TRAF2/JNK arm of ER stress may regulate autophagy through modulation of Beclin-1 function and expression. Intriguingly, it has been recently shown that XBP-1 ablation increases autophagy and protects from the toxicity induced by the aggregates of the enzyme superoxide dismutase 1 in a model of Amyotrophic lateral sclerosis [127]. These observations suggest that the XBP-1 may play a different role than TRAF2/JNK on the regulation of autophagy by the Ire1 arm of the UPR. 

#### 3.2.2. Ca^2+^ Signalling and Autophagy

ER stress activation is frequently accompanied by calcium release into the cytosol which leads to the activation of several Ca^2+^-regulated signalling pathways [116]. Different agents (including ER stress inducers) have been shown to produce an increase in cytosolic calcium concentration and activate autophagy. One of the mechanisms connecting Ca^2+^ release from the ER and autophagy is the stimulation of AMPK [128]. As explained above, several kinases regulate mTORC1 including AMPK, which inhibits mTORC1 by activating TSC2 [129]. AMPK is considered an important energy sensor that becomes activated upon ATP cellular depletion or phosphorylation by different kinases [96]. Three AMPK upstream kinases have been identified to date: LKB1, Ca^2+^/calmodulin-dependent kinase kinase *β* (CaCMKK*β*), and transforming growth factor-beta-activating kinase 1 (TAK1) [96]. Jäättelä and coworkers showed that increases in cytosolic Ca^2+^ concentration upon treatment with different ER stress inducers stimulate CaMKK*β*, leading in turn to AMPK activation, inhibition of mTORC1, and autophagy stimulation [128]. The same group has recently shown that TRAIL-induced autophagy is also mediated by AMPK, in this case through a mechanism that involves phosphorylation of AMPK by TAK1 and not by LKB1 or CaM-KK*β* [130]. These observations suggest that AMPK may play an important role in the regulation of autophagy in response to different Ca^2+^-dependent and independent stress signals. 

Another Ca^2+^-activated kinase that regulates autophagy in response to ER stress is the death associated protein kinase 1 (DAPK). DAPK is a Ser/Thr kinase that plays an important role as tumor suppressor due to its ability to promote apoptosis and autophagy [131]. Thus, DAPK-deficient MEFs are less sensitive to ER stress-induced autophagy than their wild-type counterparts [132]. Activation of DAPK upon ER stress relies on the dephosphorylation of an inhibitory autophosphorylation site of the kinase by a PP2A phosphatase [132], which suggests that additional ER stress-activated signals (apart from Ca^2+^ release) are required to stimulate the proautophagic activity of the kinase. Regarding the mechanisms by which DAPK regulates autophagy, it has been recently shown that DAPK phosphorylates Beclin-1 on the BH3-only domain preventing thus the interaction of this protein with Bcl-2 [133, 134]. In addition, DAPK regulates p53 in a p19Arf-dependent manner [135]. As p53 modulates autophagy through different mechanisms [112, 136–138], this could be another way by which DAPK could regulate autophagy in response to certain ER stress stimuli.

The protein kinase C theta (PKC*θ*) has been also implicated in regulating autophagy in response to ER stress in a calcium-dependent manner. Thus, knock-down of PKC*θ* (but not inactivation of the UPR signalling routes) prevented autophagy as induced by acute ER stress [139]. In this study, inactivation of mTORC1, under the used concentrations of thapsigargin, thapsigargin were not observed, which suggests that different signalling routes may converge in the regulation of autophagy under ER stress situations involving calcium mobilization [140]. 

Another link between Ca^2+^, ER stress, and autophagy relies on the modulation of the inositol 1,4,5-trisphosphate receptor (IP3R). This receptor releases Ca^2+^ from ER stores in response to different cellular signals, although it could also play additional functions derived from its ability to interact with different proteins, including members of the Bcl-2 family [141]. Inhibition of the IP3R with xestospongin B [108] or lithium-induced decrease of myo-inositol-1,4,5-triphosphate (IP3) levels [142] promotes autophagy. Intriguingly, these effects seem to be independent of the Ca^2+^ mobilization function of IP3R [143]. Thus, it has been recently shown that use of pharmacological inhibitors of the IP3R disrupts the interaction of this protein with Beclin-1 [144] which could be an additional way of regulating the pro-autophagic function of this protein. Further investigation is nevertheless necessary to clarify whether this mechanism participates in the activation of autophagy in response to ER stress.

### 3.3. Survival or Death after Autophagy Stimulation by ER Stress

As discussed for the case of ER stress, autophagy is currently considered a cell survival mechanism that, under certain cellular settings, can also promote cell death. Consequently, depending on whether pharmacological or genetic inhibition of autophagy enhances or prevents cell death, activation of autophagy after ER stress has been assigned respectively a cytotoxic [75, 115, 119, 132, 145–147] or a protective [5, 6, 115, 119, 128] role. It is worth noting that depending on the intensity of the stimulus, the cell type (normal versus cancer cells), and the cellular context, (hypoxia, starvation, treatment with antitumoral agents, or presence of mutations) the final outcome of autophagy activation could be different.

An important problem at the time of predicting whether induction of ER stress will activate autophagy in a protective or cytotoxic way is our relative lack of understanding of the molecular mechanisms through which autophagy regulates cell death. Thus, autophagy has been proposed to protect from apoptosis, operate as an alternative cell death mechanism (e.g., in cells that are defective in apoptosis), or act upstream of apoptosis to activate this cellular process, (reviewed in [133, 148]). As discussed in the previous section, some of the key regulatory steps in the activation of autophagy upon stimulation of ER stress (such as mTORC1 inhibition or the interaction of Beclin-1 with Bcl-2) can also receive signals derived from different inputs including those not directly related with ER stress. Moreover, some of the regulatory proteins transmitting these signals such as Akt, AMPK, DAPK, or JNK play also a major role in the modulation of cell survival independently of autophagy. It is therefore essential to consider the cellular context in order to understand how the different ER stress signals are integrated to yield a protective or cytotoxic autophagic response.

## 4. ER Stress and Autophagy in Anticancer Therapy: A Double-Edged Sword

**Figure 3:**
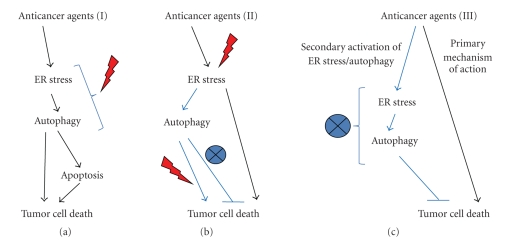
Hypothetic therapeutic strategies based on the modulation of ER stress and autophagy. Different strategies involving modulation of ER stress and autophagy could be potentially used in antitumoral therapies. A. One type of antitumoral agents (e.g., cannabinoids) activates ER stress and autophagy as a mechanism to promote cancer cell death. In these cases, strategies aimed at increasing the stimulation of ER stress and autophagy might be beneficial; B. Other anticancer agents (e.g., PDT) activate ER stress as part of the mechanisms by which they promote cancer cell death. Secondary ER stress-induced activation of autophagy may contribute to cell death (in apoptosis-deficient cells) or to cell survival (in apoptosis competent cells). Thus, depending on the tumor features, autophagy inhibitors or inducers might be administered to improve the response to these anticancer agents. C. A third type of antitumoral agents (e.g., Imatinib mesilate) activates a protective ER stress/autophagy response secondarily to its primary antitumoral mechanism. Inhibition of ER stress and/or autophagy would help to reduce the resistance to this type of therapy.

From the above discussion, it is clear that ER stress and autophagy can activate both prosurvival mechanisms as well as lethal programs, especially under conditions of enduring ER stress and organellar damage. Thus activation of the UPR and autophagy may either impede or facilitate drug-mediated cell killing, and it is plausible that this will depend on the type of cancer and cytotoxic agents used. While a growing number of reports have started to identify molecular elements of the cross-talk between ER stress and autophagy (see Section 3.2), thus unraveling potential druggable targets, knowledge of the functional outcome of the activation of these pathways in cancer cells responding to chemotherapeutics is still very limited. In terms of therapeutic outcome, drugs (or a combination thereof) capable of activating the proapoptotic branch of the UPR while simultaneously inhibiting its prosurvival function should provide the highest therapeutic benefit. Moreover, if autophagy activated following ER stress is a survival response restoring ER homeostasis (e.g., by the removal of protein aggregates), its pharmacological blockage could protract UPR activation until a critical threshold is reached, which may precipitate its proapoptotic function. On the other hand, autophagy may endorse the proapoptotic functions of certain ER stress pathways (see also Section 4.1) or become a lethal backup pathway in cancer cells with defect on apoptotic signaling [133, 148]. (Figure 3).

A wide array of conventional and experimental chemotherapeutic agents has been shown to stimulate ER stress and activation of UPR along with autophagy in cancer cells. For example, tunicamycin, thapsigargin, and brefeldin A activate autophagy in colon and prostate cancer cells thus mitigating ER stress and protecting against cell death. However, autophagy induced by the same chemicals does not confer protection in a normal human colon cell line and in the nontransformed murine embryonic fibroblasts but rather contributes to cell death [115]. The combined administration of Vorinostat (a histone deacetylase inhibitor) and Sorafenib (a tyrosine kinase inhibitor) to carcinoma cells promotes cell death although activates at the same time a protective ER stress-driven autophagic response [149]. Similarly, the resistance to Imatinib mesylate (a BCR/ABL tyrosine kinase inhibitor used for the treatment of chronic myeloid leukaemia) might also rely—at least in part—on the secondary activation of ER stress-induced autophagy [150]. By contrast, cannabinoid treatment activates ER stress and autophagy leading to apoptotic cell death of glioma and pancreatic cancer cells but not of nontransformed embryonic fribroblasts or primary astrocytes (in which neither ER stress nor autophagy is activated in response to the treatment with these compounds) [75]. Likewise, other agents such as Nelfinavir (an HIV protease inhibitor with anticancer activity) [151, 152] or Melanoma differentiation associated gene-7/interleukin 24 (mda-7/IL-24) [145, 153] activate an ER stress response that promotes autophagy and apoptosis of cancer cells. Increased expression of Tetraspanins (a family of proteins that facilitate the spatial organisation and localisation of multiprotein complexes in distinct membranal microdomains) has also been shown to activate ER stress and autophagic cell death [154].

Understanding the precise molecular mechanisms that regulate the extent of autophagy activation in response to different triggering signals as well as the ones that control the interplay of this cellular process with apoptosis is therefore crucial to design new antitumoral therapies based on the modulation of the ER stress-autophagy response. Here we discuss further a selected group of clinically used or promising cytotoxic drugs with a demonstrated ability of inducing both UPR and autophagy, as paradigms to discuss the potential of targeting these pathways in cancer therapy. 

### 4.1. Cannabinoids

Cannabinoids, the active components of marijuana, of which THC is the most important owing to its high abundance and potency [155], exert a wide variety of biological effects by mimicking endogenous substances, the endocannabinoids, that bind to and activate specific cannabinoid receptors [156]. Cannabinoids are currently being investigated as potential antitumoral agents. Thus, treatment with these agents has been shown to curb tumor growth in various animal models of cancer [157–159]. The antitumoral action of cannabinoids is based on the ability of these agents to inhibit tumor angiogenesis and activate apoptosis of cancer cells [157, 158]. 

Our recent findings have unravelled that cannabinoids induce autophagy in different types of tumor cells, including glioma/astrocytoma and pancreatic cancer cells, whereas they do not activate this cellular process in nontransformed cells (which are resistant to the cell death-promoting activity of cannabinoids) [75]. Of interest, pharmacological or genetic inhibition of autophagy prevented cannabinoid-induced cell death as well as apoptosis, whereas abrogation of apoptosis prevented cell death but not autophagy as induced by these agents. These observations led us to conclude that induction of autophagy is part of the mechanism by which cannabinoids promote the apoptotic death of cancer cells. The in vivo relevance of these findings was demonstrated by the observation that cannabinoid treatment reduced tumor growth and activated autophagy and apoptosis in subcutaneous tumor xenografts derived from human U87MG astrocytoma cells and transformed mouse embryonic fibroblasts (MEFs). Likewise, similar results have been obtained in an orthotopic model of pancreatic cancer, in which we had previously shown a proapoptotic and an antitumoral action of cannabinoids ([122, 123], Salazar, M. and Velasco G., unpublished observations). Furthermore, autophagy-deficient tumors (generated by subcutaneous injection of transformed Atg5^−/−^ MEFs) were resistant to THC antitumoral action, strongly supporting that autophagy is essential for the antineoplastic activity of cannabinoids. In addition, analysis of samples obtained from two glioblastoma multiforme patients indicated that THC administration might also trigger autophagy-mediated cell death in human tumors [75].

As discussed in the previous section, the mechanism responsible for the activation of autophagy upon THC administration relies on a cannabinoid receptors-induced early accumulation of de novo-synthesized ceramide [an event that takes place in the ER [160]], which leads in turn to ER dilation and increased eIF2*α* phosphorylation [75]. Activation of this ER stress response induces the up-regulation of several genes, including the stress-regulated protein p8 and its downstream targets ATF-4 and CHOP and the pseudokinase TRB3, which are required for the stimulation of autophagy in response to cannabinoid action. TRB3 plays a crucial role in the induction of autophagy upon THC administration through its inhibitory interaction with Akt, which leads in turn to mTORC1 inhibition. In agreement with these observations, treatment of mice with THC decreased mTORC1 activity, stimulated autophagy and apoptosis, and reduced tumor growth in xenografts generated with p8^+/+^ cells but not in those generated with p8^−/−^ cells (in which TRB3 is not up-regulated in response to THC [122]), further confirming that the p8/TRB3 pathway plays an essential role in the activation of autophagy and cell death by cannabinoids also in vivo. 

Cannabinoids activate therefore a cell death-promoting signalling route that involves the stimulation of ER stress, autophagy, and apoptosis in cancer cells. Thus, cannabinoids constitute an interesting tool to investigate the differential molecular mechanisms that are responsible for the stimulation of autophagy-mediated cell death. On the other hand, the selectivity of cannabinoids (which only stimulate the above-described cell death promoting pathway in cancer cells) together with a low toxicity and good safety profile makes of these agents promising antineoplastic tools.

### 4.2. Photodynamic Therapy

Photodynamic therapy (PDT) is an anticancer therapy involving the selective photosensitization of malignant cell types, usually involving porphyrins, porphyrin analogs or other agents with suitable photophysical properties. The initial step in the photodynamic process involves localization of the photosensitizing agent at subcellular loci, followed by irradiation with visible light of the appropriate wavelength [161, 162]. This results in formation of singlet oxygen and other ROS that can cause photodamage at sites where the photosensitizing agent has localized. Since singlet oxygen will not migrate more than a fraction of a micron from the site of formation, as a result, photodamage can be quite specific. Thus, a distinguished property of PDT is that ROS formation is mainly targeted to a particular subcellular site and affects a rather specific subset of molecular targets. PDT with various agents has been shown to induce apoptosis along with autophagy and, in most cases, autophagy is activated as a mean to protect cells from killing [8]. Agents found to be clinically useful were reported to show affinity for the ER, mitochondria, lysosomes, or combinations of these sites [163]. A well-studied paradigm of ER-localizing dye is hypericin, a naturally occurring phototoxin with promising applications in bladder cancer [164]. Consistent with its predominant reticular localization in cultured cells [82], light activation of hypericin is coupled with massive ER expansion, preceding ultrastructural features of apoptosis, both in vitro and in bladder cancer bearing rats (Verfaillie, T. and Agostinis, P. unpublished observations), and stimulation of UPR [165]. UPR activation is likely the result of immediate ROS-damage to the SERCA pump, depleting ER-Ca^2+^ store, which is followed by the concomitant activation of autophagy and mitochondrial apoptosis [82]. This ROS paradigm of ER stress is linked to a persistent activation of the PERK-eIF2*α*-CHOP axis, with proapoptotic function (Verfaillie, T. and Agostinis, P. unpublished observations). Induction of autophagy in ER stressed cells unable to mount an apoptotic response (because of *bax/bak* deficiency) results in increased photokilling, suggesting the activation of an “autophagic cell death” pathway [82]. Conversely, in apoptosis-competent cells, blocking autophagy stimulation following ER stress by siRNAs that target essential modulators of the autophagic machinery, sensitizes to cell death, thus revealing a cytoprotective role for this pathway (Dewaele, M. and Agostinis, P. unpublished results). Hence, it is tempting to speculate that autophagy inhibition may potentiate the proapoptotic PERK pathway resulting in a better therapeutic opportunity, only when the cancer cell's apoptotic machinery has not been fully disabled. Further studies are required to establish whether suppression of the autophagic pathway along with UPR stimulation may represent a valuable therapeutic strategy in hypericin-based PDT.

### 4.3. Proteasome Inhibitors

Targeting proteasomal degradation has proven to be a valuable approach in various cancer treatments, and proteasome inhibitors have emerged as a new class of ER stress agents. Moreover, recent evidence suggests that when used in combination with certain cytotoxic drugs, such as PDT, proteasomal inhibitors are capable of enhancing their anticancer efficacy, making these agents a very promising class of pharmacological agents in combinatorial therapy.

#### 4.3.1. Bortezomib

Bortezomib (PS-341 or Velcade) distinguishes itself from other proteasome inhibitors as it specifically inhibits the 26S proteasome by selectively blocking its chymotryptic activity. Velcade has been clinically approved for treatment of multiple myeloma and mantle cell lymphoma [166, 167] and has been shown to successfully induce apoptosis in various human cancer cell lines including myeloma, prostate, and breast cancers as well as squamous cell carcinoma [168–170]. Moreover, preclinical studies indicate that bortezomib displays anticancer activity against pancreatic cancers [171], one of the most aggressive human diseases. One of the potential mechanisms underlying the apoptotic effects of bortezomib in cancer cells relies on its ability to inhibit the NF-*κ*B pathway by blocking the degradation of its cytoplasmic inhibitor I*κ*B*α* [172]. However, inhibition of NF-*κ*B alone could not fully account for the antitumor effect by bortezomib, suggesting additional pathways being involved [173]. This additional pathway turned out to be dependent on ER stress. Since proteasomal degradation of misfolded proteins retrotranslocated from the ER to the cytosol represents the final step in ERAD, proteasomal inhibition causes an additional burden of unfolded proteins on the ER. This explains the high efficacy of bortezomib treatment against types of cancer cells in which the ER is already predisposed with a considerable load. For instance, hypoxic cancer cells that otherwise show increased resistance to genotoxic agents as well as myeloma cells producing high amounts of immunoglobulins are hypersensitive to treatment with proteasome inhibitors [174, 175]. Therefore, therapies that target the ER response in combination with bortezomib ought to be more successful. Indeed, it was shown that bortezomib sensitized pancreatic cancer cells to ER stress mediated apoptosis [176]. Additionally, we recently found a significant retardation of tumor growth in vivo in two different murine tumor models when photofrin-based PDT, a PDT approach stimulating the UPR, was combined with bortezomib, or other clinically used proteasome inhibitors [177]. This suggests that blocking the proteasome might offer a new therapeutic avenue to potentiate the antitumor effect of PDT. 

Interestingly, Schewe and Aguirre-Ghiso [178] found that myeloma cells surviving bortezomib treatment attenuated eIF2*α* phosphorylation and induction of CHOP. Combined treatment with the GADD34-PP1 complex inhibitor salubrinal restored eIF2*α* phosphorylation and CHOP induction, maximizing bortezomib induced apoptosis, thus suggesting that strategies capable of sustaining CHOP expression might be required to successfully eradicate tumors. 

Aside from proteasomal degradation, autophagy represents another important mechanism for degrading intracellular material. Furthermore, these processes are functionally coupled and proteasomal inhibition has been shown to stimulate autophagy, likely as a compensatory mechanism [119]. Surprisingly, whether autophagy enhances apoptosis induced by proteasomal inhibitors or not seems to depend on whether the treated cells are transformed or not [179]. These findings suggest that a combined inhibition of both cellular degradation systems would enhance the antitumoral efficacy. Indeed, autophagy was shown to be activated in MCF-7 cells treated with bortezomib, by a mechanism which involved proteasomal stabilization of ATF4 and ATF4 dependent upregulation of LC3B. This mechanism was suggested to contribute to the resistance of breast cancer cells towards bortezomib [180]. However, a recent study wherein myeloma cells where treated with bortezomib in combination with the autophagy inhibitor 3-methyl adenine (3-MA) resulted in an antagonistic response instead of the expected synergizing effect [181].

## 5. Conclusions

Research during the last decade has contributed to highlight the important role of ER stress and autophagy in the maintenance of the cellular homeostasis. The last few years have also evidenced that both processes are closely related as some of the signalling routes activated during the ER stress response are involved in stimulating autophagy. Intriguingly, activation of autophagy after ER stress can be either protective of cytotoxic. For example, accumulation of unfolded proteins in neurodegenerative diseases may activate a protective autophagy response. By contrast induction of ER stress in cancer cells may promote the stimulation of autophagy-mediated cell death or the activation of a protective autophagy that may contribute to the resistance to certain antitumoral therapies. Thus, different factors such as the intensity of the ER stress signal, the simultaneous activation of additional pathways, the cell type, and so forth, must be integrated to yield a specific autophagic response. Considering that escape from drug-mediated cell killing is one of the major obstacles of current cancer therapy, a better understanding of the role played by these processes in cancer cells in response to chemotherapy would help us to devise new and more efficient therapeutic opportunities utilizing inhibitors or activators of these ER stress pathways.

## Abbreviations

ARE: Antioxidant Response Element
ASK-1: Apoptosis Signal-regulating Kinase 1
ATF4: Activating Transription Factor 4
ATF6: Activating Transription Factor 6
Atg: Autophagy Gene
Bad: Bcl-2 Antagonist of Cell Death
Bak: Bcl-2 antagonist/killer
Bax: Bcl2-Associated X Protein
Bcl-2: B-cell lymphoma 2
BH3: Bcl-2 Homology 3
BI-1: Bax Inhibitor 1
Bim: Bcl2-interacting mediator of cell death
BiP: Immunoglobulin heavy chain-binding protein
CHOP: CAAT/enhancer binding protein (C/EBP) homologous protein
DAPK: Death Associated Protein Kinase
EDEM-1: ER Degradation-Enhancing *α*-Mannosidase-Like Protein
eIF2*α*: eukaryotic Initiation Factor-2*α*
ER: Endoplasmic Reticulum
ERO1: ER Oxidase1
ERSE: ER Stress Response Element
GADD34: Growth Arrest and DNA Damage-Inducible Gene 34
HO-1: Heme Oxygenase 1
Hsp: Heat shock protein
IP3R: Inositol 1,4,5- Trisphosphate Receptor
IRE1: Iiositol Requiring Enzyme 1
JNK: c-Jun N-terminal Kinase
LC3: Microtubule-associated protein light chain 3
MAPK: Mitogen Activated Protein Kinase
NF-*κ*B: Nuclear Factor *κ*B
PERK: RNA dependent protein kinase (PKR)-like ER kinase
PP1: Protein Phosphatase 1
PP2A: protein phosphatase 2A
Puma: P53 upregulated mediator of apoptosis
ROS: Reactive Oxygen Species
SERCA: Sarco/Endoplasmic Reticulum Ca^2+^ ATPase
TRAF2: TNF (Tumor Necrosis Factor) receptor associated factor 2
TRB3: Tribbles homologue 3
UPR: Unfolded Protein Response
UPRE: UPR Response Element
XBP1(u/s): X-box Binding protein 1(unspliced/spliced).
